# Complete mitochondrial genome and the phylogenetic position of the Pawak croaker *Pennahia pawak* (Perciformes: Sciaenidae)

**DOI:** 10.1080/23802359.2017.1334526

**Published:** 2017-06-21

**Authors:** Bai-An Lin, Chang-Chang Guo, Min Liu

**Affiliations:** College of Ocean and Earth Sciences, Xiamen University, Xiamen City, China

**Keywords:** Bayesian tree, mitogenome, Pennahia, Perciformes, phylogenetic relationship, Sciaenidae

## Abstract

In this study, the complete mitogenome of the Pawak croaker *Pennahia pawak* was first determined. This mitogenome is 16,408 bp in length, and consists of 37 genes with the typical gene order and direction of transcription in vertebrates. The overall nucleotide composition is: 27.7% A, 29.5% C, 15.9% G, and 26.9% T. Sizes of the 22 tRNA genes range from 66 to 75 bp. One start codons (ATG) and two stop codons (AGA and TAA/TA/T) were detected in 13 protein-coding genes. In the Bayesian tree based on the complete mitogenomes of 17 species (including *P. pawak*) from the family Sciaenidae, all nodes were strongly supported. The phylogenetic results suggested that *P. pawak* has the closest relationship to the silver croaker *P. argentata*, a species from the same genus.

The family Sciaenidae (Perciformes) is commonly known as croakers and drums. As an economically important group of fishes, it comprises about 270 species in 70 genera in the world (Nelson 2006). The Pawak croaker *Pennahia pawak* (Lin 1940) occurs in western Pacific, inhabiting along coastal waters from southern China to Gulf of Thailand and southern Indonesia (Sasaki 2001). The species feeds mainly on small crustaceans with approximate maximum total length of 25 cm (http://www.fishbase.org). In this study, we presented the complete mitochondrial genome of *P. pawak* and assessed its phylogenetic relationship based on another 16 available mitogenomes in the family Sciaenidae with one available mitogenome in the family Epinephelidae and two in the family Polynemidae selected as an outgroup.

One specimen of *P. pawak* (BBW20170222) was collected by a bottom trawler in the Gulf of Tonkin, Guangxi Province, China. The protocol and data analysis methods followed Chen et al. (2014). The complete mitochondrial genome of *P. pawak* is 16,408 bp in length (GenBank accession number: KY978753) with the typical gene order and transcriptional direction in vertebrates. It contains two rRNA genes, 22 tRNA genes, 13 protein-coding genes, and one control region. The overall nucleotide composition is as follows: 27.7% A, 29.5% C, 15.9% G, and 26.9% T. In the 13 identified protein-coding genes, only one start codons (ATG) were detected. Two stop codons (AGA and TAA/TA/A) were found; *COX1* was terminated by the AGA codon, and the other 12 protein-coding genes by either the TAA or incomplete T or TA codon that may form complete termination signal UAA via post-transcriptional polyadenylation (Ojala et al. 1981). The 12S (875 bp) and 16S (1711 bp) rRNA genes are located between the tRNA-*Phe* and tRNA-*Leu1* genes, separated by the tRNA-*Val* gene. The lengths of 22 tRNA genes range from 66 to 75 bp; 21 tRNAs can be folded into the typical cloverleaf secondary structures with the exception of tRNA-*Ser2* in which the DHU arm was replaced by a simple loop. A 36 bp inserted sequence was identified as the putative origin of L-strand replication (OL). The control region was 822 bp in length with high A + T (63.0%) and low G + C (37.0%) composition.

Published mitogenomes of all 17 species of the family Sciaenidae (including *P. pawak* in this study) together with *Cephalopholis boenak* from the family Epinephelidae, and *Eleutheronema tetradactylum* and *Polydactylus sextarius* from the family Polynemidae were used to assess the phylogenetic relationship of *P. pawak*. Phylogenetic tree was constructed with the partitioned Bayesian method based on the dataset combined by three partitions (the alignments of the 1, 2 codon positions of 12 H-strand encoded protein-coding genes together with two rRNAs) under the GRT + I+G model (Ronquist & Huelsenbeck 2003). As the phylogenetic tree showed, all nodes were strongly supported with high value of posterior probability (Figure 1). The result shows that *P. pawak* was placed as sister to the silver croaker *P. argentata* of the same genus, which is consistent to the croaker phylogenetic results using combined mitochondrial and nuclear genomes (Lo et al. 2015).

**Figure 1.**
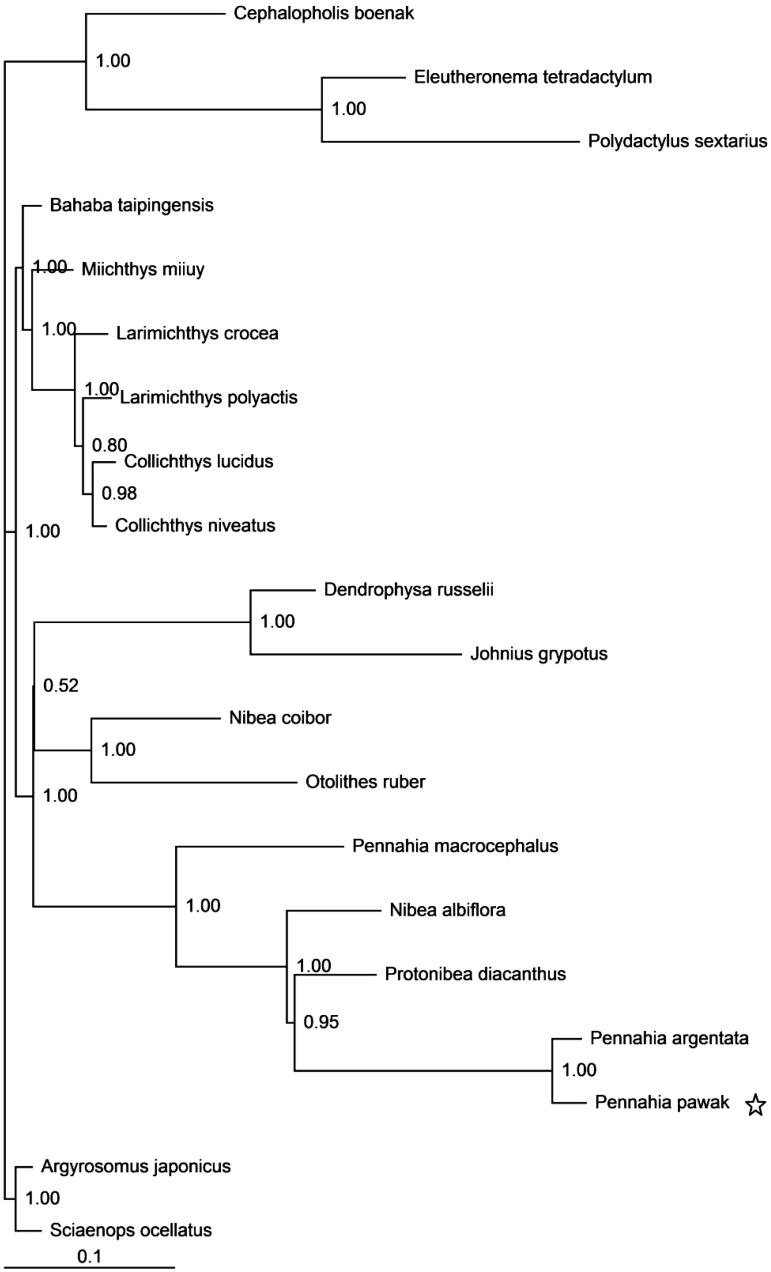
Phylogenetic position of the Pawak croaker *Pennahia pawak*. *Cephalopholis boenak* (KC537759.1), *Eleutheronema tetradactylum* (KC878730.1), and *Polydactylus sextarius* (NC_027088.1) were selected as the outgroup. The other 17 species from the family Sciaenidae are: *Argyrosomus japonicus* (NC_017610.1), *Bahaba taipingensis* (NC_018347.1), *Chrysochir aureus* (NC_016987.1), *Collichthys lucidus* (JN857362.1), *Collichthys niveatus* (JN678726), *Dendrophysa russelii* (NC_017606.1), *Johnius grypotus* (KC491206), *Larimichthys crocea* (NC_011710.1), *Larimichthys polyactis* (GU586227.1), *Miichthys miiuy* (NC_014351.1), *Nibea albiflora* (NC_015205.1), *Nibea coibor* (NC_025307.1), *Otolithes ruber* (KX929060), *Pennahia argentata* (KC545800.1), *Pennahia macrocephalus* (NC_031409.1), *Protonibea diacanthus* (NC_024573.1), and *Sciaenops ocellatus* (NC_016867.1).
